# Dibenzyl Disulfide Adsorption on Cationic Exchanged Faujasites: A DFT Study

**DOI:** 10.3390/nano9050715

**Published:** 2019-05-08

**Authors:** Etienne Paul Hessou, Miguel Ponce-Vargas, Jean-Baptiste Mensah, Frederik Tielens, Juan Carlos Santos, Michael Badawi

**Affiliations:** 1 Laboratoire de Physique et Chimie Théoriques, Faculté des Sciences et Technologies, CNRS, Université de Lorraine, Boulevard des Aiguillettes, 54500 Vandoeuvre-lès-Nancy, France; tiganahess@gmail.com; 2 Laboratoire de Chimie Théorique et de Spectroscopie Moléculaire, Université d’Abomey-Calavi, 03 BP 3409 Cotonou, Benin; menfolben@yahoo.fr; 3 Institut de Chimie Moléculaire de Reims, Université de Reims Champagne-Ardenne, 51687 Reims, France; miguel.ponce-vargas@univ-reims.fr; 4 Chemistry (ALGC), Vrije Universiteit Brussel, Pleinlaan 2, B-1050 Brussel, Belgium; 5 Laboratorio de Corrosión, Departamento de Ciencias Químicas, Facultad de Ciencias Exactas, Universidad Andres Bello, Av. República 330, 8370186 Santiago, Chile

**Keywords:** ab initio, zeolite, faujasite, copper, silver, alkali metals, sulfur compounds, DBDS

## Abstract

Although dibenzyl disulfide (DBDS) is used as a mineral oil stabilizer, its presence in electrical transformer oil is associated as one of the major causes of copper corrosion and subsequent formation of copper sulfide. In order to prevent these undesirable processes, MY zeolites (with M = Li, Na, K, Cs, Cu or Ag) are proposed to adsorb molecularly DBDS. In this study, different MY zeolites are investigated at the DFT+D level in order to assess their ability in DBDS adsorption. It was found that CsY, AgY and CuY exhibit the best compromise between high interaction energies and limited S-S bond activation, thus emerging as optimal adsorbents for DBDS.

## 1. Introduction

Mineral insulating oil, which is conventionally divided in aliphatic, naphthenic and aromatic hydrocarbons, is widely used in transformers, circuit breakers and other electrical equipment. However, there is usually still a small amount of sulfur containing compounds present, which are obtained from the refining technique or that have been added as antioxidants, which could cause the corrosion of copper coils within the transformers [1,2,3,4,5,6,7,8,9]. Dibenzyl disulfide (DBDS) is one of the most abundant and representative of these sulfur species. Thus, the development of new adsorbents to improve the DBDS selective removal is of paramount importance. In this regard, various types of adsorbents, such as, metal oxides, clays, activated carbon and zeolites have been used as adsorbents for organic compounds [10,11,12,13]. Among them, cation-exchanged zeolites are attractive for this application due to their thermal stability, facility to be separated from the reaction products, ability to be regenerated, high surface area, size-selective adsorption property and capacity to include a large variety of cationic sites. These materials have also been evaluated as adsorbents for sulfur compounds [11,12,13].

Particularly, ion exchanged faujasites (type Y zeolite) have proven to be efficient adsorbents for dimethyl disulfide (DMDS) [14], hydrogen sulfide (H_2_S) [15,16,17,18], tetrahydrothiophene (THT) and t-butyl mercaptan (TBM) [19,20]. In the same vein, systems using Ag^+^, Cu^2+^ and Ce^3+^ exchanged Y zeolites have been employed for adsorption of thiophenes from organic liquid medium [21]. Moreover, adsorbent systems based on Cu-Y, Ag-Y and Ce-Y have been used for DBDS removal from insulating oil with good performance [1]. Indeed, AgY was able to remove 76% of DBDS, 80% of CeY and 97% of CuY. However, adsorption enthalpies were not measured, and these results deserve to be explained at a molecular level. Therefore, for the sake of this study the faujasite zeolite was retained to examine the DBDS adsorption.

The adsorption efficiency of the cation exchanged zeolites had been attributed to an adsorption mechanism involving π complexation of cations in zeolites with sulfides after ion exchange, but some questions have not been answered yet, such as the structural effect of the adsorbent (type of zeolite), the amount and type of metal exchanged on the zeolite. As the time required to perform cationic exchange can be very long, here we propose to undertake a theoretical screening of all monovalent cations that can be experimentally incorporated in the Y zeolite.

Density functional theory (DFT) is one of the most effective tools to elucidate adsorption processes at molecular level, and is conveniently employed to investigate such processes in various cation-exchanged zeolites [22,23,24,25,26,27,28,29]. Therefore, in the present work a systematic DFT investigation will be performed in order to evaluate the use of metal-exchanged faujasites as adsorbents of dibenzyl disulfide (DBDS). The paper is organized as follows. Section 2 contains the computational details. In Section 3, the DBDS properties are analyzed, first in gas phase and then adsorbed in NaY, and finally, the interaction is studied in different exchanged zeolites LiY, NaY, KY, CsY, Cu(I)Y and Ag(I)Y. In Section 4, the main conclusions are summarized.

## 2. Methodology

In this study, density functional theory was used applying the augmented plane wave method (PAW) [30] to describe the electron-ion interactions with a cut-off energy of 450 eV. The functional of Perdew Burke Ernzerhof (PBE) [31] was employed, and the Kohn–Sham equations were solved self-consistently until the energy difference of the cycles is less than 10^−6^ eV. The atomic positions were fully optimized until all forces were smaller than 0.01 eV/Å per atom. Due to the large size of the unit cell, all computations were performed only at the Γ-point. The computations were performed using the Vienna Ab initio Simulation Package (VASP) [32].

To accurately describe the adsorption process of DBDS molecule in the zeolite, van der Waals (vdW) interactions, which are not included in the PBE functional were taken into account [23,28,33,34] using two correction schemes, D2 and TS/HI, with each method representing a different degree of complexity in the correction formulation. In the D2 correction of Grimme [35,36,37] the vdW interactions are described as an atom-pairwise correction and the C_6_ coefficients are defined for each atomic species irrespective of the system. On the other hand, in the Tkatchenko–Scheffler scheme with iterative Hirshfeld partitioning (TS/HI) [38,39,40], the C_6_ coefficients are dependent on the electron density and the ionic character of the system. Both methods were recently implemented in VASP code [37,39,40]. Once the total energy of the different systems has been computed with either of the two dispersion correction schemes, the interaction energies of DBDS over cation-exchanged FAU at 0 K are obtained using Equation (1) below.

(1) $$∆E_{\mathrm{int}}={\;E}_{\mathrm{FAU}-X}-{\;E}_{\mathrm{FAU}}-{\;E}_{X}\;,$$

where

- E_FAU-X_: the energy of the system containing faujasite with DBDS;
- E_FAU_: the energy of the faujasite alone;
- E_X_: the energy of the isolated DBDS in gaseous phase.

In a similar fashion, the contribution of dispersion forces $∆E_{\mathrm{disp}}$ to the interaction energy was computed as:

(2) $$∆E_{\mathrm{disp}}={\;E}_{\mathrm{disp}\;\mathrm{FAU}-X}-{\;E}_{\mathrm{disp}\;\mathrm{FAU}}-{\;E}_{\mathrm{disp}\;X}\;.$$

Finally, a Noncovalent Interactions Analysis (NCI), as proposed by Yang and coworkers, was conducted by using the NCI code [41,42] to describe more precisely the different types of noncovalent interactions such as π-cation. The suitability of this methodology to explain host-guest interactions in metal-containing systems has been reported previously [42,43].

The faujasite is a three dimensional network belonging to the family of large pore zeolites. Its framework is composed of sodalite cages also named β cages with diameter of 6.6 Å connected to supercages also named α cages and having a diameter of 12.4 Å. These two units are interconnected by hexagonal prisms whose opening is of 2.3 Å (D6R). These supercages are linked together by a 12MR ring with diameter of 7.4 Å, forming the porous accessible network.

**Figure 1:**
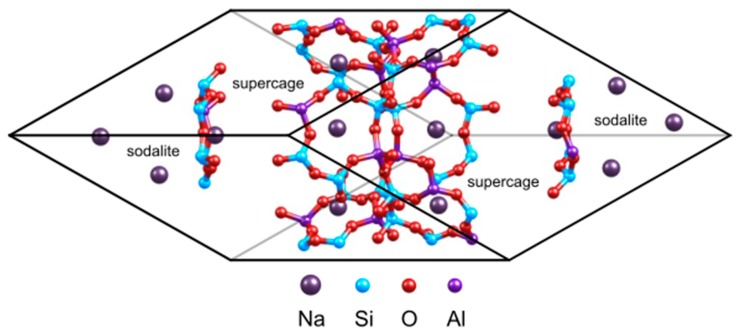
Unit cell of the sodium exchange faujasite.

The siliceous structure of faujasite crystallizes within the *Fd*3*m* symmetry space group [44]. The lattice parameters of the standard cubic cell (576 atoms, Si_192_O_384_) are: a = b = c = 25.028 Å [45,46]. In the present study, in order to reduce the computational cost, we consider the primitive rhombohedral cell containing 144 atoms (Figure 1). The primitive cell of faujasite contains two supercages and eight hexagonal windows connecting the sodalite with the supercage. In this work, we exchange 14 Si by Al atom inside this primitive cell (see Figure 1), resulting in a Si/Al ratio of 2.43 which corresponds to Y zeolite [47]. Therefore, the molecular formulas of the investigated cells are M_14_Al_14_Si_34_O_96_, with M = Li^+^, Na^+^, K^+^, Cs^+^, Cu^+^ or Ag^+^. As the cation volumes influence the cell parameters, we carried out a full relaxation of the cells for each system investigated at the PBE+D2 level of theory.

## 3. Results

### 3.1. DBDS in Gas Phase

**Figure 2:**
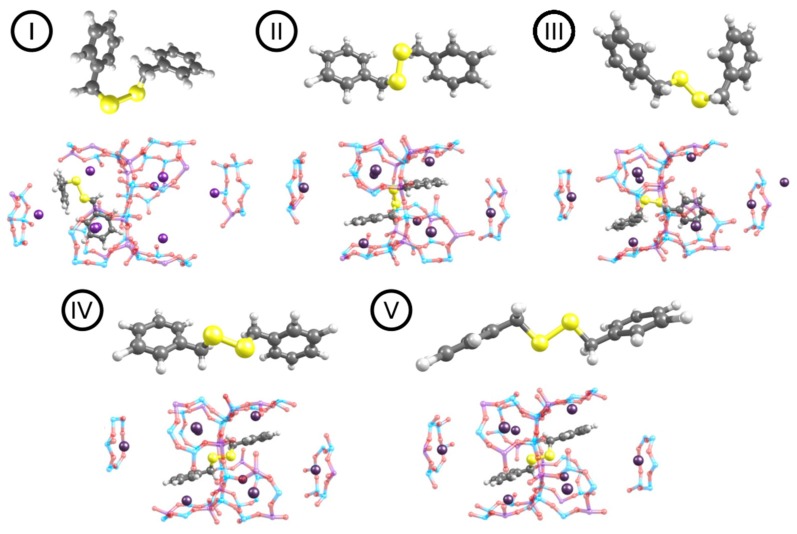
For each DBDS conformer **I**–**V**: Optimized DBDS structure before (up)/after (down) the incorporation in the Y zeolite (here shown for the case of NaY). In I and II, the DBDS phenyl rings keep the gas configuration inside the cell, whereas in II, IV and V they adopt a parallel configuration. Given its higher energy, structure V is not further considered in this work.

The conformation analysis of DBDS in the gas phase reveals that the conformer I is the most stable one followed by conformer II, III, IV and V (see Figure 2). These results are in agreement with those obtained by Saavedra et al. [7] who also found a series of closely related conformers with a C-S-S-C dihedral angle of about 90°. The main difference between the most stable and the least stable conformer is the relative orientation of the aromatic rings described by this C-S-S-C dihedral angle. In conformer I the aromatic rings are almost perpendicular to each other whereas in conformer V they are almost parallel. Conformer V was found to be the least stable (see Table 1) and it will not be further considered in this work. Another plausible difference between the conformers is the disulfide bond length, which varies within 0.7 Å (see Table 1). The energy difference between the different conformers I to IV was calculated to be within a range of around 13 kJ mol^−1^ at both PBE+D2 (Table S1) and PBE+TS/HI (Table 1) levels of theory. Both approaches predict conformer I (C-S-S-C dihedral angle of about 90°) to be the most stable of the five DBDS conformers. Conformer II, less stable than conformer I by 10.3 kJ mol^−1^ was found in solid phase by Lee et al. [48] while conformer IV, less stable than conformer I by 13.4 kJ mol^−1^ was obtained by Meichning et al. [49]. At this point we can conclude that there is no significant difference in structural parameters obtained at both levels of theory, i.e., PBE+D2 (Table S1 after the references) and PBE+TS/HI (see Table 1).

### 3.2. DBDS Adsorbed in NaY (Si/Al = 2.43)

Subsequently, the four most stable DBDS gas phase conformations (I to IV) were considered for the study of DBDS adsorption in the NaY zeolite. In almost all cases, the conformation geometries were altered after adsorption, as a consequence of the rise of π-cation interactions between the sodium cations and the aromatic rings, except for I where the initial conformation already allowed an effective DBDS-zeolite coupling (Figure 2).

In order to investigate the adsorption of DBDS within the Y zeolite, a NCI analysis was carried out. This enables to clarify the role of cation-π interactions between the DBDS molecule and the exchangeable cations in the faujasite structure. This analysis is based on a 2D plot of the reduced density gradient *s*, and the electron density *ρ*, where *s* can be expressed as:

(3) $$s=\frac{1}{2{\left(3\pi^{2}\right)}^{1/3}}\frac{\left|\nabla \rho \right|}{\rho^{4/3}}.$$

A noticeable variation in *s* generates density critical points leading to an isosurface which reveals the presence of non-covalent interactions. The second eigenvalue of the electronic density Hessian (λ_2_) enables us to determine the nature of such interactions; hence, intense stabilizing forces are characterized by λ_2_ < 0, steric repulsion by λ_2_ > 0, and weak interactions by λ_2_ ≈ 0. These λ_2_ values are represented by using a color scale.

**Figure 3:**
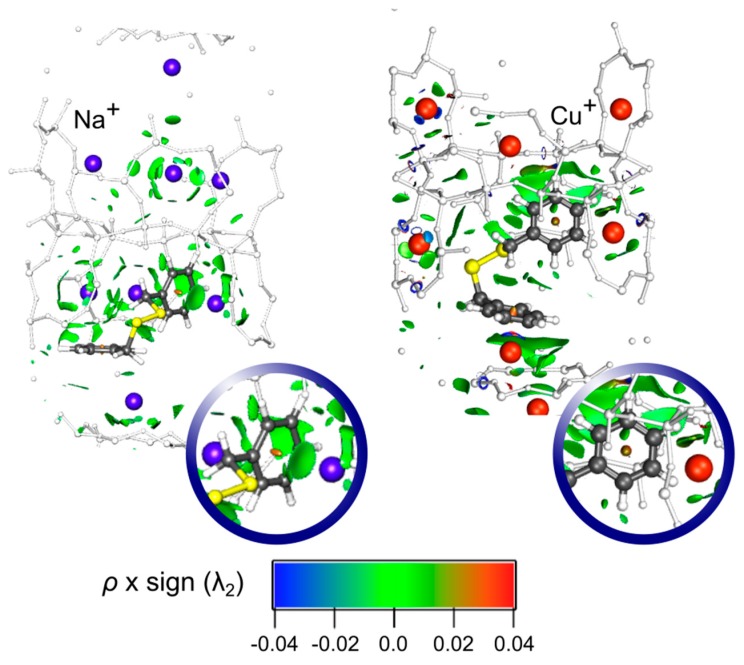
Noncovalent Interactions Analysis (NCI) analysis for the conformer I of the NaY and CuY faujasites containing DBDS. An isosurface of 0.01 a.u. was considered.

The NCI results for the conformer I of DBDS embedded in NaY and CuY systems are presented in Figure 3. In the DBDS-NaY structure an isosurface is clearly observed between one DBDS aromatic ring and a Na^+^ ion, revealing an efficient cation-π interaction in this case. Conversely, the absence of this isosurface in the analog DBDS-CuY, along with a shorter distance between the second aromatic ring and a Cu^+^ (2.350 Å) in comparison to DBDS-NaY (3.815 Å) suggest that a covalent character interaction is the main factor governing the DBDS inclusion in CuY, prior to the rise of noncovalent forces. Remarkably, neither of the two systems exhibits an isosurface between the cation and sulphur, suggesting a negligible interaction between the disulfide bridge and the Na^+^/Cu^+^ ions. Hence, the DBDS-CuY adsorption complex formation is an advantageous situation in order to avoid the copper sulfide generation.

### 3.3. Cationic Screening Results for DBDS Adsorption in Cu(I)-Exchanged Y Zeolites

The interaction energies of the DBDS conformers (I to IV) with LiY, NaY, KY, CsY, Cu(I)Y and Ag(I)Y were computed at the PBE+D2 (Table S3) and PBE+TS/HI (Table 2) levels of theory.

Both methods give similar trends. However, it can be noticed that the use of D2 leads to higher interaction energies (in absolute value) for zeolites containing large cations such as CsY—around 100 kJ mol^−1^ higher in absolute value—in comparison to those obtained using the TS/HI dispersion method. Remarkably, for other small molecules in the same zeolite structure [28], D2 was found to overestimate the adsorption enthalpies compared to TS/HI.

It is clear from the interaction energies that the most stable gas phase conformation does not adsorb stronger to MY (see Table 2). This is in line with the fact that most stable structures are the least reactive. Conformer II was found the best starting conformation for adsorption for Na^+^, K^+^, Cs^+^, Ag^+^ cations and conformer IV for Li^+^ and Cu^+^ cations. The adsorption conformation does not differ significantly from the gas phase conformations II or IV. Conformer II is always the most favorably adsorbed, except in LiY and Cu(I)Y where IV exhibits the highest interaction energy. The interaction energies range between −170 and −275 kJ mol^−1^ for LiY and CuY, respectively.

The interaction energy between DBDS and MY for M = alkali metal increases with the cation softness and decreases with their electronegativity, in agreement with the chemical concepts developed by Pearson et al. [50,51]. The interaction energy also increases in line with the cation radius. Remarkably, the trends for the group X ions (Cu and Ag) are opposite to those observed for the alkali cations, as in this case the interactions are not only electrostatic.

### 3.4. Evaluation of the Regenerability of the Materials

**Figure 4:**
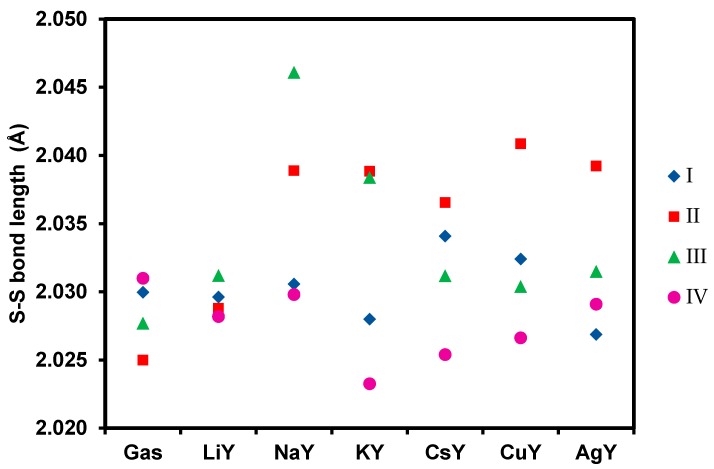
S-S bond length of DBDS conformers in gas phase and upon adsorption.

Formation of byproducts is undesirable and can lead to the deactivation of the sorbent [52,53]. Therefore, a molecular adsorption of DBDS is targeted, and its dissociation into two benzylsulfides must be prevented. To this end, the stretching of the S-S bond of DBDS upon adsorption in the MY zeolites was evaluated. The results obtained are reported in Figure 4 and Table S5.

Regarding the conformer I, the analysis of the S-S bond lengths before and after adsorption reveals an almost negligible evolution of the S-S bond upon adsorption (less than 0.02 Å). Hence, conformer I is not expected to dissociate upon adsorption over cationic-exchanged faujasites, except for CsY. In the case of the conformer II, an elongation of the S-S bond is observed for all cations. With regard to the variation of the S-S bond after adsorption of the conformer III, one can notice a lengthening of the S-S bond with all the cations, which is more pronounced in NaY.

Regarding the conformer IV, the least stable of the four conformers studied, one can observe a shortening of the S-S bond for all cations, making them good candidates for DBDS removal. We highlight the fact that Li^+^ and Cu^+^ exchanged zeolites give the highest interaction energies with values of −170.0 and −275.1 kJ mol^−1^, respectively.

As the proportion of each conformer cannot be controlled experimentally, especially under the conditions of electric transformers, we have to select a Y zeolite formulation where the S-S bond activation would be the most limited as possible. According to this criterion, NaY has to be rejected because it could decompose the conformers II and III quite easily, while LiY appears as an ideal candidate. Hence, CsY, AgY and CuY can be selected as a compromise between high interaction energies and limited S-S bond activation. These recommendations are partially supported or in agreement with the experimental results obtained by Wan et al. [1], who found that CuY was an efficient material to remove DBDS (97% of removal after 2 h), followed by AgY (76%). Unfortunately, we do not have other elements of comparison as adsorption enthalpies were not measured in this study [1].

## 4. Conclusions

The adsorption of DBDS on MY zeolites (with M = Li, Na, K, Cs, Cu or Ag) was investigated using periodic DFT including dispersion corrections in order to propose an efficient DBDS adsorbent for its removal from mineral insulating oils. From the different MY structures herein studied, it was clear that DBDS adsorbs via its phenyl moieties instead of forming a sulfur bond. Among the five conformers, conformer I is recognized as the most stable one followed by conformers II, III and IV that are less stable however equivalent, while conformer V is the least stable and was not considered in this study. The interaction energies of the four considered conformers range from −170 to −275 kJ mol^−1^ in the cationic-exchanged Y zeolites investigated in the present work.

In the case of DBDS-CuY, the comparative NCI analysis with the analog DBDS-NaY, and the shorter distance between the DBDS phenyl group and a Cu(I) belonging the supercage, suggest a covalent character interaction, explaining the high calculated adsorption energies found for this system. Remarkably, neither DBDS-NaY nor DBDS-CuY exhibit NCI isosurfaces between the cation and sulphur, suggesting a negligible interaction between them. 

In general, one can conclude that the adsorption complex (DBDS/MY) displays an advantageous geometry in order to avoid copper sulfide formation. Moreover, CsY, AgY and CuY can be selected as a compromise between high interaction energies and limited S-S bond activation, and are predicted to be optimal adsorbents for molecular DBDS, avoiding the formations of dissociated species. In the future we plan to extend our study on other zeolites and MOFs, still on the DBDS molecule, but also its sub-species and other sulfated species that may be present in the oil, such as C_6_H_5_-CH_2_-SH and SH_2_ in the frame of ab initio molecular dynamics.

## Figures and Tables

**Table 1:** Selected structural parameters and energies of the dibenzyl disulfide (DBDS) structures optimized with PBE+TS/HI. Energies in kJ mol^−1^, distances in Å, and dihedral angles in degrees.

Dibenzyl Disulfide Conformers	Structural Parameters in Gas Phase		ΔE_rel_ (kJ mol^−^^1^)
	d(S-S) (Å)	ang(C-S-S-C) (°)	
I	2.030	90.9	0.0
II	2.025	85.5	10.3
III	2.028	84.7	11.1
IV	2.031	86.5	13.4
V	2.104	179.9	46.7

**Table 2:** Computed (PBE+TS/HI) total interaction energies ∆E_int_ and the corresponding contributions of dispersion energies of the four conformers of DBDS with LiY, NaY, KY, CsY, CuY and AgY. Energies in kJ mol^−1^.

CONFORMERS								
	I		II		III		IV	
	ΔE_int_	ΔE_disp_	ΔE_int_	ΔE_disp_	ΔE_int_	ΔE_disp_	ΔE_int_	ΔE_disp_
LiY	−144.5	−84.8	−158.2	−91.5	−159.2	−95.4	−170.0	−126.7
NaY	−180.4	−130.5	−194.7	−128.6	−160.9	−129.2	−187.1	−130.1
KY	−188.6	−133.4	−207.8	−137.6	−184.8	−135.3	−189.6	−134.4
CsY	−210.9	−170.5	−236.9	−180.7	−210.6	−160.2	−230.1	−167.6
CuY	−217.2	−110.8	−262.0	−123.8	−238.9	−123.6	−275.1	−127.2
AgY	−234.0	−131.5	−251.3	−131.2	−222.7	−123.5	−236.3	−163.3

## Supplementary Materials

The following are available online at https://www.mdpi.com/2079-4991/9/5/715/s1, Figure S1. Dibenzyil disulfide (DBDS) structures optimized with Gaussian (starting from the DBDS structures optimized with Vienna Ab initio Simulation Package (VASP)). Figure S2. Evolution of the interaction energy as a function of the variation of the S-S distance after adsorption. Table S1. Selected structural parameters of the optimized dibenzyil disulfide structures. Energies in kJ mol^−1^, distances in Å and angles in degrees. Table S2. Selected structural parameters and energies of the DBDS structures optimized with Gaussian (starting from the DBDS structures optimized with PBE+D2). Energies in kJ mol^−1^, distances in Å and angles in degrees. Table S3. Computed (PBE+D2) total interaction energies ∆Eint of the four conformers of DBDS with LiY, NaY, KY, CsY, CuY and AgY. The contributions of dispersion energies to the interaction energies ∆Edis are reported in this table in parentheses. Energies in kJ mol^−1^. Table S4. Selected structural parameters of dibenzyil disulfide inside the Na-faujasite cell. Energies in kJ mol^−1^, and distances in Å. Table S5. S-S bond (Å) before and after adsorption upon LiY, NaY, KY, CsY, CuY and AgY.
