# Supplementary material for: Dibenzyl Disulfide Adsorption on Cationic Exchanged Faujasites: A DFT Study
Source: Nanomaterials (Basel). 2019 May 8;9(5):715. doi: 10.3390/nano9050715 (PMC6566968; doi:10.3390/nano9050715)
Supplement: Supplementary file 1 [file nanomaterials-09-00715-s001.pdf]

nanomaterials

Supplementary Information

Dibenzyl Disulfide Adsorption on Cationic Exchanged Faujasites: A DFT Study

Etienne Paul Hessou [1,2], Miguel Ponce-Vargas [3], Jean-Baptiste Mensah [2], Frederik Tielens [4,*], Juan Carlos Santos [5,*] and Michael Badawi [1,*]

1 Laboratoire de Physique et Chimie Théoriques, Faculté des Sciences et Technologies, CNRS, Université de Lorraine, Boulevard des Aiguillettes, 54500 Vandoeuvre-lès-Nancy, France; tiganahess@gmail.com
2 Laboratoire de Chimie Théorique et de Spectroscopie Moléculaire, Université d'Abomey-Calavi, 03 BP 3409 Cotonou, Benin; menfolben@yahoo.fr
3 Institut de Chimie Moléculaire de Reims, Université de Reims Champagne-Ardenne, 51687 Reims, France; miguel.ponce-vargas@univ-reims.fr
4 Chemistry (ALGC), Vrije Universiteit Brussel, Pleinlaan 2, B-1050 Brussel, Belgium; frederik.tielens@vub.be
5 Laboratorio de Corrosión, Departamento de Ciencias Químicas, Facultad de Ciencias Exactas, Universidad Andres Bello, Av. República 330, 8370186 Santiago, Chile
* Correspondence: frederik.tielens@vub.be (F.T.); jsantos@unab.cl (J.C.S.); michael.badawi@univ-lorraine.fr (M.B.); Tel.: +333 7274 9867 (M.B.)

I	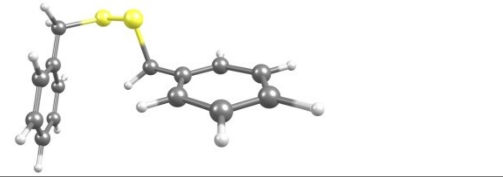
II	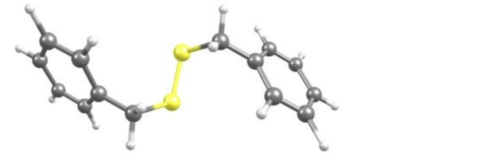
III	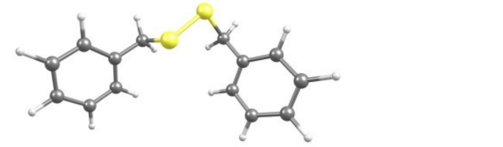
IV	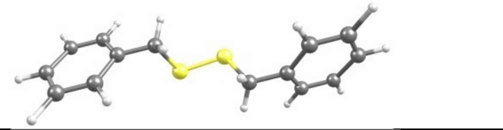
V	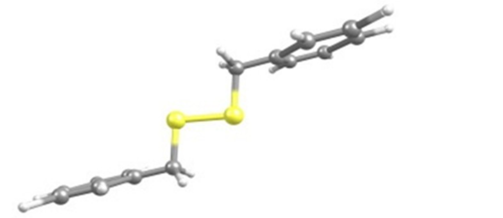

Figure S1. Dibenzyil disulfide (DBDS) structures optimized with Gaussian (starting from the DBDS structures optimized with Vienna Ab initio Simulation Package (VASP)).

Table S1. Selected structural parameters of the optimized dibenzyil disulfide structures. Energies in kJ/mol, distances in Å and angles in degrees.

**DBDS conformers**	**Optimization of the gas phase molecule starting from its structure before the incorporation in the cell**		**ΔE-rel**	**ΔE-reorg**	**Inside the Na-faujasite cell**		**ΔErel**
	**d(S-S)**	**C-S-S-C**			**d(S-S)**	**C-S-S-C**	
**I**	**2.029**	**88.1**	0.0	10.6	2.03	95.4	0.0
II	2.023	84.3	10.8	13.6	2.04	91.2	13.8
III	2.028	83.9	11.2	21.7	2.05	65.9	22.3
IV	2.032	85.8	12.3	12.4	2.03	103.4	14.1
V	2.105	179.9	44.7	–22.6	2.04	103.6	11.5

Table S2. Selected structural parameters and energies of the DBDS structures optimized with Gaussian (starting from the DBDS structures optimized with PBE+D2). Energies in kJ/mol, distances in Å and angles in degrees.

**Dibenzyl disulfide**	**Structural parameters in gas phase**		**ΔE-rel**
	**d(S-S)**	**C-S-S-C**	
**I**	2.079	92.1	1.7
II	2.074	85.3	3.4
III	2.077	86.7	1.7
IV	2.080	87.5	0.0
V	2.080	87.5	0.0

In contrast, the plane waves calculations give a larger range of magnitude in ΔErel regarding the DBDS conformers.

Table S3. Computed (PBE + D2) total interaction energies ΔE_{int} of the four conformers of DBDS with LiY, NaY, KY, CsY, CuY and AgY. The contributions of dispersion energies to the interaction energies ΔE_{dis} are reported in this table in parentheses. Energies in kJ/mol.

**DBDS Conformers**	**LiY**	**NaY**	**KY**	**CsY**	**CuY**	**AgY**
I	–156.5<br>(–112.9)	–195.3<br>(–156.0)	–176.1<br>(–122.5)	–345.4<br>(–295.8)	–237.6<br>(–130.9)	–252.0<br>(–157.0)
II	–183.0<br>(–135.8)	–202.5<br>(–148.5)	–195.4<br>(–130.0)	–357.9<br>(–299.4)	–265.7<br>(–148.5)	–275.6<br>(–166.0)
III	–188.6<br>(–147.9)	–178.5<br>(–166.3)	–176.5<br>(–134.9)	–321.5<br>(–271.2)	–262.8<br>(–159.0)	–253.4<br>(–179.2)
IV	–194.8<br>(–176.0)	–193.1<br>(–150.2)	–187.7<br>(–142.9)	–344.0<br>(–281.8)	–288.2<br>(–149.2)	–249.7<br>(–219.8)

Table S4. Selected structural parameters of dibenzyil disulfide inside the Na-faujasite cell. Energies in kJ/mol, and distances in Å.

**Structure**	**π-cation distance**	**ΔE_{int}**	**ΔE_{disp}**
**I**	2.465/2.561	–195.3	–156.0
**II**	2.757/2.784	–202.5	–148.5
**III**	2.497/2.843	–178.5	–166.3
**IV**	2.526/2.711	–193.1	–150.5
**V**	2.600/2.665	–225.5	–151.3

Table S5. S-S bond (Å) before and after adsorption upon LiY, NaY, KY, CsY, CuY and AgY.

	**Gas**	**LiY**	**NaY**	**KY**	**CsY**	**CuY**	**AgY**
I	2.030	2.030	2.031	2.028	2.034	2.032	2.027
II	2.025	2.029	2.039	2.039	2.037	2.041	2.039
III	2.028	2.031	2.046	2.038	2.031	2.030	2.032
IV	2.031	2.028	2.030	2.023	2.025	2.027	2.029

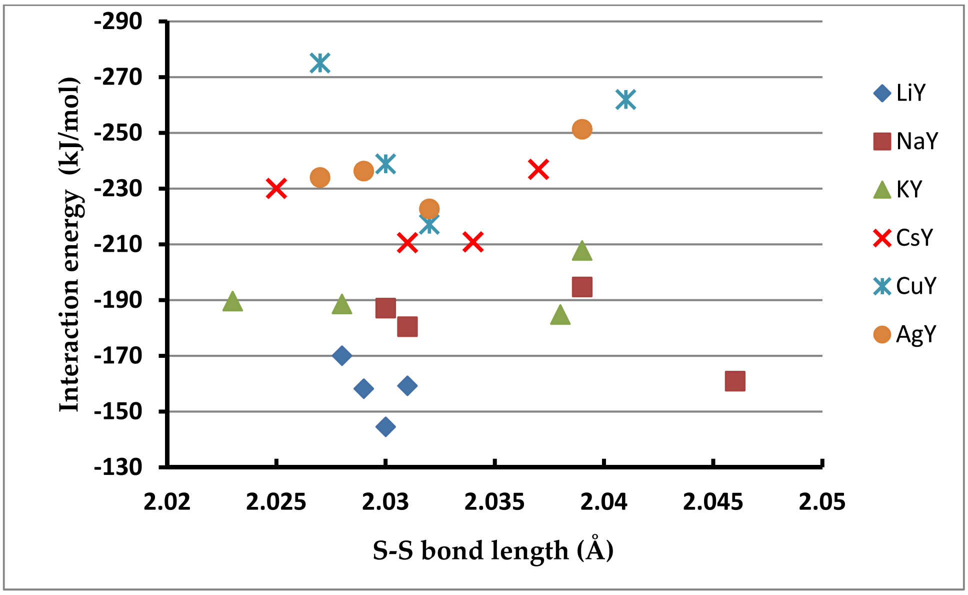

Figure S2. Evolution of the interaction energy as a function of the variation of the S-S distance after adsorption.

When considering the Figure S2 which shows the variation of the interaction energy as a function of the length of the S-S bond, it is noted that the Cu^{+} cation seems to be the first which gives significant interaction energy without bond activation (–275.1 kJ / mol).
